# Development and validation of a nomogram predicting pathologic complete response to neoadjuvant chemotherapy in luminal type breast cancer patients using MammaPrint® and clinicopathological factor

**DOI:** 10.1371/journal.pone.0356871

**Published:** 2026-09-21

**Authors:** Sarah Al safi, Tae Kyung Yoo, Jisun Kim, Il Yong Chung, Beom Seok Ko, Hee Jeong Kim, Jong Won Lee, Byung Ho Son, Sae Byul Lee

**Affiliations:** 1 Department of Surgery, Al Adan Hospital, Hadiya, Ministry of Health, Kuwait; 2 Division of Breast Surgery, Department of Surgery, University of Ulsan College of Medicine, Asan Medical Center, Seoul, Republic of Korea; Weill Cornell University, UNITED STATES OF AMERICA

## Abstract

**Purpose:**

Pathological complete response (pCR) of neoadjuvant chemotherapy (NACT) is the most important predictor of successful treatment in locally advanced breast cancer and is associated with survival outcomes. The aim of our study is to investigate a validated nomogram that predicted MammaPrint® to assess the response rate in luminal type breast cancer.

**Methods:**

This study retrospectively included patients from Asan Medical Center, Seoul, South Korea who were diagnosed with luminal type breast cancer and received NACT and underwent breast cancer surgery from August 2008 to December 2021. We used a nomogram previously published by colleagues at our center, using a cutoff score of 183. The primary outcome of this study was to investigate the use of nomogram adopted from a 70-gene MammaPrint for prediction of pCR following NACT.

**Results:**

Data from 1,574 patients were collected. The nomogram using MammaPrint predicted pCR (odds ratio 2.092, 95% confidence interval 1.507–2.905; p < 0.001). Based on multivariate logistic regression analysis, estrogen receptor status, progesterone receptor status, clinical tumor stage, clinical node stage, and Ki67 index were independent predictive factors of pCR in the primary cohort. The area under the curve of the validated nomogram was 0.64 (95% confidence interval, 0.60–0.67), indicating the nomogram predicted pCR in the neoadjuvant setting.

**Conclusion:**

This study provides evidence that the adopted nomogram using the MammaPrint risk assessment based on clinicopathological pre-NACT factors can be utilized in luminal type breast cancer to aid in treatment decisions.

## Introduction

Understanding the immunophenotype of breast cancer tailored the treatment towards patient and tumor specific characteristics. Neoadjuvant chemotherapy (NACT) has been increasingly used in locally advanced breast cancer [1]. The most important predictor for a successful neoadjuvant treatment is measured by pathological complete response (pCR) [2,3]. Pathological complete response is defined by the absence of residual invasive cancer following completion of neoadjuvant systemic therapy (ypT0N0 or ypTisN0,; AJCC 8^th^ edition) [Ref]. Achieving pCR following NACT varies across the different subtypes of breast cancer [4,5].

Luminal type breast cancer is the most common subtype accounting for 50–60% of cases. It has an overall favorable prognosis, but the response to NACT is the lowest with pCR achieved in 18% of cases. Triple negative and HER2 positive tumor subtypes have higher rates of achieving pCR after NACT at 50% and 30% respectively [6]. Despite the low response rate in luminal breast cancer, NACT can be offered in certain cases such as large tumor size >5 cm, > 4 positive nodes or in cases of desiring breast or axilla conserving [7,8]. There has been an ongoing controversy regarding a clear selection criteria for NACT in luminal type breast cancer [9].

In the neoadjuvant setting, it is difficult to discern who will benefit from NACT and achieve pCR. Therefore, the development of a safe, accurate, and non-invasive pre NACT tool for predicting pCR in patients with luminal type breast cancer eligible for NACT is essential [10,11]. There are tools used in the adjuvant setting to assess the requirement for adjuvant treatment in early luminal breast cancer such as Mammaprint®. It classifies them as high or low risk for distant recurrences. Most patients with HR-positive/HER2-negative cancer based on MammaPrint results have a low risk. In the MINDACT trial, a phase III randomized controlled trial, Mammaprint identified low risk patients with high clinical risks who have excellent outcomes without chemotherapy [12]. Additionally, MammaPrint index was positively associated with achieving pCR (p < 0.001) in the NBRST study by Whitworth et al. [13]. The ISPY2 trial examined the utility of Mammaprint in HR positive HER2 negative breast cancer. They found that patients who were MammaPrint High2 were more chemosensitive, with a higher rate of pCR of up to 23.0% compared to 6.1% in those who were MammaPrint High1 [7].

Therefore, developing a tool to identify this subgroup of patients during preoperative assessment is important. In the present study, due to the low pCR rate in our institution, we aimed to investigate the use of a validated nomogram adopted from a 70-gene MammaPrint genomic risk assessment to assess the NACT response rate for patients with luminal type breast cancer to enable patient screening prior to initiating NACT.

## Methods

### Patient selection and variable selection

We conducted a retrospective single center study in Seoul Asan Medical Center, South Korea. From our electronic medical database, we selected patients ≥18 years of age with pathologically confirmed luminal type breast cancer that is HR-positive/HER2 negative who received NACT followed by breast cancer surgery between August 2008 and December 2021. Patients with clinical tumor stage 1–3)cT1-3) and clinical node stage 0–2 (cN0-2) were included (New table). Clinicopathological characteristics at baseline prior to NACT were obtained from the patient’s electronic medical records. It included age at diagnosis, histologic grade, nuclear grade, estrogen and progesterone receptors Allred score, Ki67 proliferative index percentage and type of breast and axillary surgery. Additionally, we have included pathological T and N stage and need for adjuvant radiotherapy. We further categorized the pathological stage based on the response to chemotherapy in terms of pCR. We used the RECIST criteria [14] to compare the response to NACT between patients classified as clinical responders, who displayed a complete response, and non-clinical responders, who displayed a partial response, stable disease, or progressive disease. Patients were classified as a complete responder if they demonstrated pCR (ypT0) or ductal carcinoma *in situ* (ypTis) in the post-operative pathology report. Further information were gathered in regards to recurrence data and death data. Patients were excluded from the study if they did not fulfil the above criteria or were missing medical information. Since it is a retrospective study, the excluded patients already received treatment in the form of NACT and breast cancer surgery. Furthermore, we employed a previously constructed nomogram (S1 Fig) published by Hwang et al. [15] that predicted the MammaPrint risk score using only four clinicopathological factors (age, nuclear grade, PR status, and Ki-67). There was no access to information that could identify individual participants during or after data collection.

The study protocol was reviewed and approved by the Institutional Review Board of Asan Medical Center (IRB 2022−0282). Informed consent was waived because the study was based on retrospective clinical data. Informed consent was waived because the study was based on retrospective clinical data and data were analyzed anonymously

### Endpoints

The primary endpoint measure to investigate the use of the nomogram adopted from MammaPrint is based on pre-treatment clinicopathological characteristics for the prediction of pCR following NACT in patients with luminal breast cancer. The secondary endpoints included prognostic factors of nomogram use on disease-free survival (DFS) and overall survival (OS) using a cutoff value of 183 in patients who received NACT. The use of a cutoff value of 183 was based on previously published study by Hwang et al [15].

### Nomogram risk prediction based on MammaPrint

The nomogram predicted the MammaPrint risk of patients as highlighted in S1 Fig [15]. The nomogram was initially constructed using the training set and then validated using the validation set; the areas under the curve (AUC) were 0.82 (95% confidence interval [CI], 0.77–0.87) and 0.77 (95% CI, 0.68–0.86), respectively. To validate the risk stratification using the nomogram, the following variables were included in the model: age at diagnosis (20–100 years), nuclear grade (range, 1–3), PR Allred scores (range, 0–8), and Ki67 labelling index (0–100). The results of the MammaPrint risk assessment in 172 patients with breast cancer were used to validate the nomogram and showed an AUC of validation set 1 of 0.73 (95% CI, 0.66–0.81) with a good calibration plot. An additional validation was subsequently performed using the data of patients with T1–3 N0–1 M0 HR-positive/HER2-negative cancer who were classified into high- and low-risk groups, based on a nomogram cutoff value of 183. This cutoff point was selected based on the Youden index. Patients with <183 total points were classified as high risk, and those with ≥183 total points were classified as low risk.

### Statistical analysis

The chi-square and Fisher’s exact test were used to compare clinical responders and non-clinical responders in terms of clinicopathological characteristics and nomogram results. A multivariate logistic regression analysis was used to assess the effect of specific variables on pCR status. Cox regression analysis and Kaplan–Meier curves were constructed to assess DFS and OS. Differences in survival were tested for significance using the log-rank test. For continuous variables, hazard ratios (HRs) represent the change in hazard associated with a one-unit increase in the covariate. For categorical variables, HRs compare the hazard of each category to that of the designated reference group. The median follow-up time was calculated using the reverse Kaplan-Meier method, which accounts for censoring of follow-up times. The predictive ability of the model was measured using the AUC under the receiver operating characteristic curve. The 95% confidence interval (CI) for the area under the curve (AUC) was calculated using the bootstrap method with 1,000 resamples. All statistical analyses were performed using IBM SPSS statistics 24.0 software (IBM Corporation, Armonk, NY, USA). The threshold of statistical significance was set at a p value of <0.05, and all p values were two sided.

## Results

### Patient characteristics

**Fig 1:**
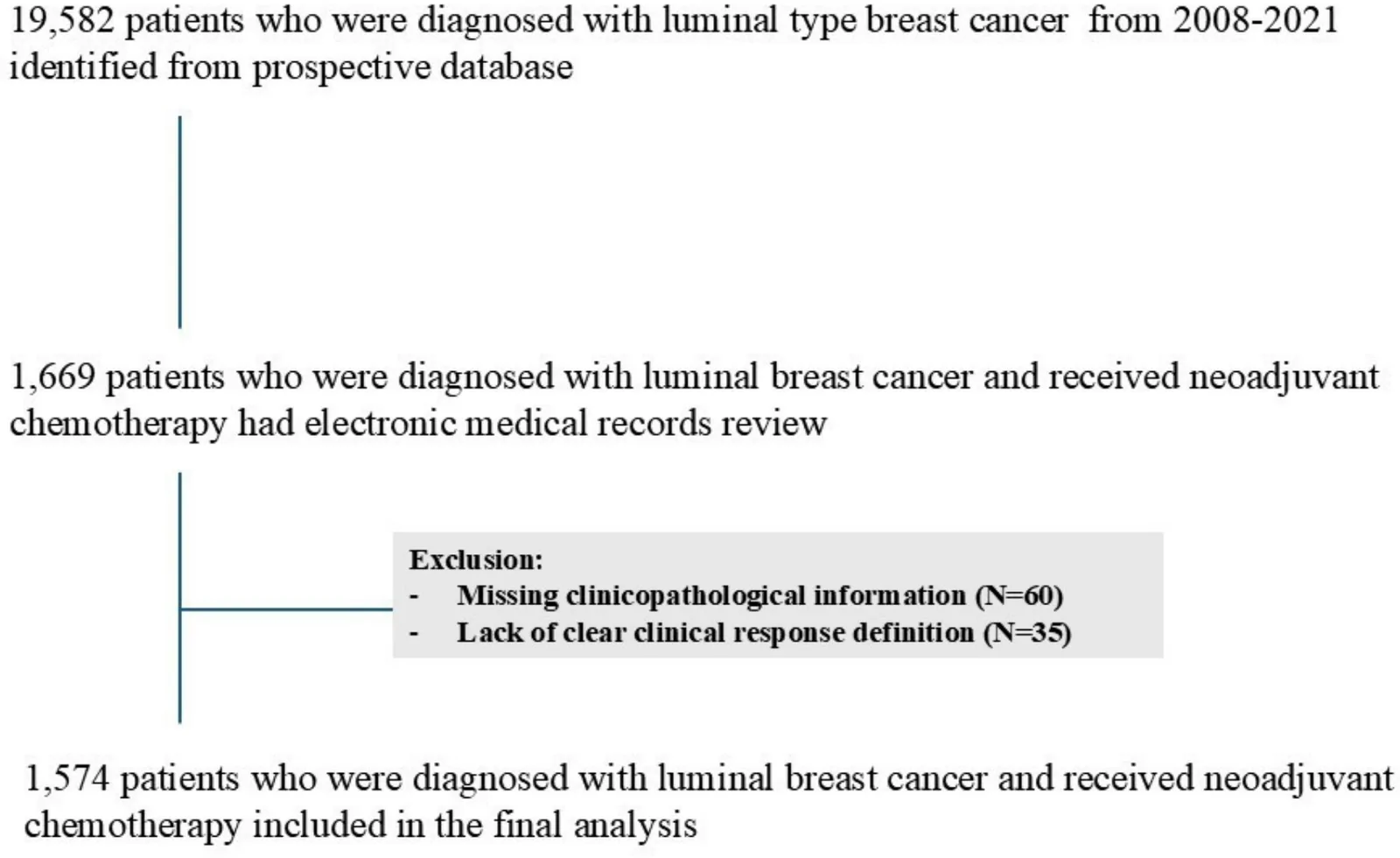
Flowchart of the participant recruitment process in the study.

We reviewed a total of 19,582 patients from our database diagnosed with luminal breast cancer from August 2008 to December 2021. A total of 1,669 patients who were diagnosed with luminal type breast cancer and received NACT from August 2008 to December 2021, but excluded 95 records due to missing information or the lack of a clear clinical response definition (Fig 1). We retrospectively analyzed the baseline characteristics of 1,574 patients included in the cohort as shown in Table 1. The analysis showed that 58.8% of patients were aged from 35 to 49 years and 31.5% were aged 50 or more years. Regarding the type of surgery, 53.6% underwent mastectomy, and the remainder underwent breast-conserving surgery. Moreover, 48.2% underwent sentinel lymph node biopsy, and 42.5% underwent both sentinel lymph node biopsy and axillary lymph node dissection. Based on the 8^th^ TNM staging system recommended by the AJCC, clinical T2 stage accounted for 65.0% of the cohort followed by cT3 (25.3%); regarding the N stage, cN1 accounted for 60.8% followed by cN0 at 31.6%. Furthermore, 92.8% of patients had invasive ductal carcinoma, whereas only 3.6% were diagnosed with invasive lobular carcinoma. Histologic and nuclear grade 2 were the most common at 81.4% and 82%, respectively. In addition, 94.3% were strongly positive for ER, and 70.5% were strongly positive for PR. Up to 2/3 of the cohort had a Ki67 index ≥ 20 (69.6%). Moreover, more than half of the cohort exhibited pCR (52.2%) followed by stable disease and complete response (24.2% and 22.2%, respectively). Finally, 1,186 patients received post-operative adjuvant radiotherapy (75.3%), and 77.1% had a nomogram score <183.

**Table 1:** Characteristics of all cohort participants (n = 1,574).

Characteristic	Values (%)
**Age at initial operation (y)**	
<35	154 (9.8)
35–49	926 (58.8)
≥50	494 (31.4)
**Type of breast surgery**	
Breast conserving surgery	731 (46.4)
Mastectomy	843 (53.6)
**Type of axillary surgery**	
Sentinel lymph node biopsy (SLNB)	758 (48.2)
Axillary lymph node dissection (ALND)	144 (9.1)
Both (SLNB and ALND)	669 (42.5)
No operation	3 (0.2)
**Clinical T stage**	
T1	153 (9.7)
T2	1023 (65.0)
T3	398 (25.3)
**Clinical N stage**	
N0	498 (31.6)
N1	957 (60.8)
N2	119 (7.6)
**Histologic subtype**	
Invasive ductal carcinoma	1461 (92.8)
Invasive lobular carcinoma	56 (3.6)
Others	57 (3.6)
**Histologic grade**	
1	37 (2.4)
2	1282 (81.4)
3	228 (14.5)
Unknown	27 (1.7)
**Nuclear grade**	
1	29 (1.8)
2	1290 (82)
3	233 (14.8)
Unknown	22 (1.4)
**Estrogen receptor status**	
Weak positive	90 (5.7)
Strong positive	1484 (94.3)
**Progesterone receptor status**	
Negative	285 (18.1)
Weak positive	179 (11.4)
Strong positive	1110 (70.5)
**Ki67 index**	
<20	453 (28.8)
≥ 20	1095 (69.6)
Unknown	26 (1.7)
**Tumor size (cm)**	
mean ± SD	2.1 ± 1.5
**Pathological nodal status**	
Negative	542 (34.4)
Positive	1032 (65.6)
**Clinical response**	
Complete response	349 (22.2)
Partial response	821 (52.2)
Stable disease	384 (24.4)
Progressive disease	20 (1.3)
**Adjuvant radiotherapy**	
Yes	1186 (75.3)
No	313 (20)
Unknown	75 (4.8)
**Nomogram**	
<183	1213 (77.1)
≧183	361 (22.9)

Y: years, T: Tumor, N: Node, SD: standard deviation.

### Patient characteristics based on response to NACT

Table 2 shows the comparison between clinicopathological characteristics of patients in the complete response and non-complete response groups. A total of 349 patients (22.1%) were defined as complete responders, whereas the majority were defined as non-complete responders (1,225 patients, 77.8%). The following variables were statistically significant: age, type of breast and axillary surgery, clinical T and N stage, histologic grade, nuclear grade, Ki67 index, pathologic T and N stages, and nomogram result. Patients in the complete response group had the following common characteristics: cT2, cN1, histologic and nuclear grade 2, strongly positive for ER and PR, and high Ki67 index. These characteristics are similar to those in the non-complete responder group. In both complete responder and non-complete responder groups, patients were more likely to be clinical T2, clinical N1, histologic and nuclear grade 2, strongly positive for ER and PR, Ki67 ≥ 20%, and receive NACT. However, patients in the complete responder group were more likely to undergo breast-conserving surgery compared to the non-complete responder group. In addition, patients in the non-complete responder group had a greater ypN1-2 compared to that in the complete responder group.

**Table 2:** Clinicopathological characteristics comparison between complete responders (CR) and non-complete responders (N = 1,574).

Characteristic	Complete response N = 349 (22.2)	Non-complete responder N = 1225 (77.8)	p-value
**Age at initial operation (y)**			0.037
<35	44 (12.6)	110 (9)	
35–49	187 (53.6)	739 (60.3)	
≥50	118 (33.8)	376 (30.7)	
**Type of breast surgery**			<0.001
Breast conserving surgery	199 (57.0)	532 (43.4)	
Mastectomy	150 (43.0)	693 (56.6)	
**Type of axillary surgery**			0.038
Sentinel lymph node biopsy (SLNB)	188 (53.9)	570 (46.5)	
Axillary lymph node dissection (ALND)	20 (5.8)	124 (10.1)	
Both (SLNB and ALND)	140 (40.1)	529 (43.2)	
No operation	1 (0.2)	2 (0.2)	
**Clinical T stage**			0.004
T1	50 (14.3)	103 (8.4)	
T2	215 (61.6)	808 (66.0)	
T3	84 (24.1)	314 (25.6)	
**Clinical N stage**			<0.001
N0	85 (24.3)	413 (33.7)	
N1	222 (63.6)	735 (60)	
N2	42 (12.1)	77 (6.3)	
**Histologic subtype**			0.171
Invasive ductal carcinoma	325 (93.1)	1136 (92.5)	
Invasive lobular carcinoma	13 (3.7)	43 (3.5)	
Others	10 (3.2)	47 (4.0)	
**Histologic grade**			
1	6 (1.7)	31 (2.5)	<0.001
2	250 (71.6)	1032 (84.2)	
3	86 (24.6)	142 (11.6)	
Unknown	7 (2.1)	20 (1.7)	
**Nuclear grade**			
1	4 (1.2)	25 (2.0)	<0.001
2	253 (72.4)	1037 (84.7)	
3	88 (25.2)	145 (11.8)	
Unknown	4 (1.2)	18 (1.5)	
**Estrogen receptor status**			<0.001
Weak positive	47 (13.5)	43 (3.5)	
Strong positive	302 (86.5)	1182 (96.5)	
**Progesterone receptor status**			<0.001
Negative	99 (28.4)	186 (15.2)	
Weak positive	55 (15.7)	124 (10.1)	
Strong positive	195 (55.9)	915 (74.7)	
**Ki67 index**			<0.001
<20	66 (19.0)	387 (31.6)	
≥20	274 (78.5)	821 (67.0)	
Unknown	9 (2.5)	17 (1.4)	
**Pathological nodal status**			<0.001
Negative	162 (46.4)	380 (31.0)	
Positive	187 (53.6)	845 (69.0)	
**Adjuvant radiotherapy**			0.133
Yes	279 (80.0)	907 (74.0)	
No	58 (16.6)	255 (20.8)	
Unknown	12 (3.4)	63 (5.2)	
**Nomogram**			<0.001
<183	300 (86.0)	913 (74.5)	
>183	49 (14.0)	312 (25.5)	

Y: years, T: Tumor, N: Node.

### Use of the nomogram as a predictive factor

**Fig 2:**
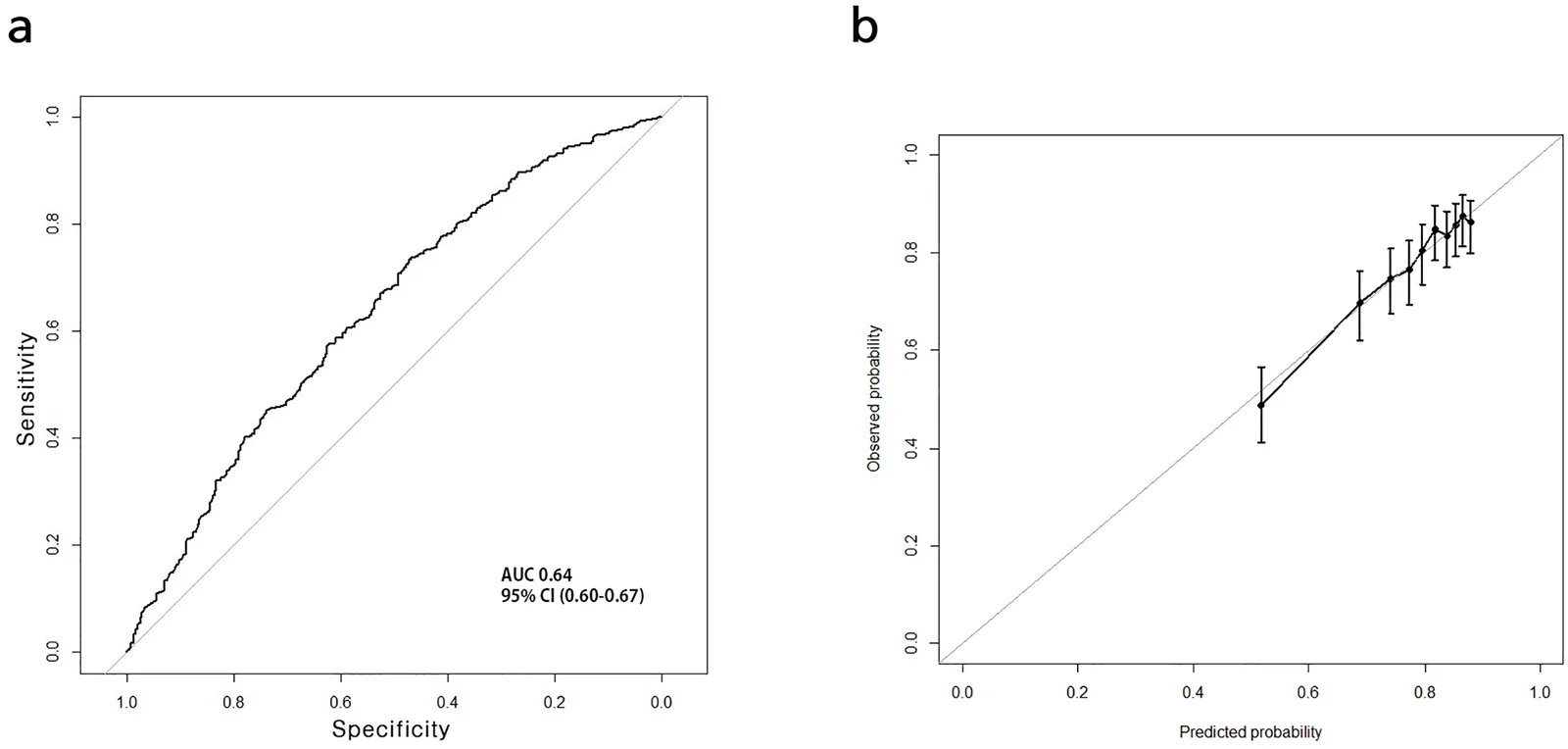
Discrimination and calibration performance of the prediction model. (a) ROC curve with AUC and 95% CI (b) Calibration plot for predicted probabilities of pCR.

The AUC of the receiver operating characteristic curve (Fig 2a) showed that the discriminative ability was acceptable (AUC, 0.64; 95% CI, 0.60–0.67), indicating that the nomogram predicted pCR in the neoadjuvant setting. Moreover, a further calibration analysis was conducted which demonstrated good agreement between predicted and observed probabilities of pCR (Fig 2b). A multivariate logistic regression analysis (Table 3) was performed to identify predictive factors of achieving pCR. The nomogram used in our study predicted pCR with a statistically significant p value of <0.0001 (odds ratio [OR], 2.092; 95% CI, 1.507–2.905). Other factors were statistically significant and associated with achieving pCR; these factors included clinical T stage (OR, 2.028; 95% CI 1.309–3.143), N stage (OR, 0.423; 95% CI, 0.264–0.677); ER-positivity (OR, 0.430; 95% CI, 0.253–0.953), PR Allred scores (OR, 0.534; 95% CI, 0.370–0.771), and Ki67 index (OR, 0.305; 95% CI, 0.125–0.743).

**Table 3:** Multivariate logistic regression analysis of total patients according to clinical response.

Variables		Odds ratio	95% CI	p-value	Coefficient
Age (y)				0.655	
	<35 (reference)	1 (ref.)			
	35–49	1.178	0.763-1.818	0.460	0.164
	≥50	0.969	0.732-1.284	0.828	−0.031
Clinical T stage				0.001	
	T1	1 (ref.)			
	T2	2.028	1.309-3.143	0.002	0.707
	T3	0.795	0.711-1.299	0.795	−0.040
Clinical N stage				0.001	
	N0	1 (ref.)			
	N1	0.423	0.264-0.677	0.0001	−0.861
	N2	0.620	0.404-0.953	0.029	−0.0478
Estrogen receptor Allred score				0.002	
	Strong positive	1 (ref.)			
	Weak positive	0.430	0.253-0.728	0.002	−0.845
Progesterone receptor Allred score				0.001	
	Strong positive	1 (ref.)			
	Negative	0.876	0.565-1.358	0.553	−0.133
	Weak positive	0.534	0.370-0.771	0.001	−0.627
Histologic grade				0.728	
	Grade 1	1 (ref.)			
	Grade 2	0.661	0.098-4.433	0.670	−0.414
	Grade 3	0.451	0.103-1.988	−.293	−0.795
Nuclear grade				0.561	
	Grade 1	1 (ref.)			
	Grade 2	1.310	0.134-12.829	0.817	0.270
	Grade 3	2.467	0.419-14.544	0.318	0.903
Ki67 index				0.003	
	<20	1 (ref.)			
	≥20	0.305	0.125-0.743	0.009	−1.188
	Unknown	0.470	0.196-1.124	0.09	−0.755
					
Nomogram				<0.0001	
	<183 (0)	1 (ref)			
	≥183 (1)	2.092	1.507-2.905	<0.0001	0.738

CI: confidence interval, Y: years, T: Tumor, N: Node.

### Use of the nomogram as a prognostic factor

**Fig 3:**
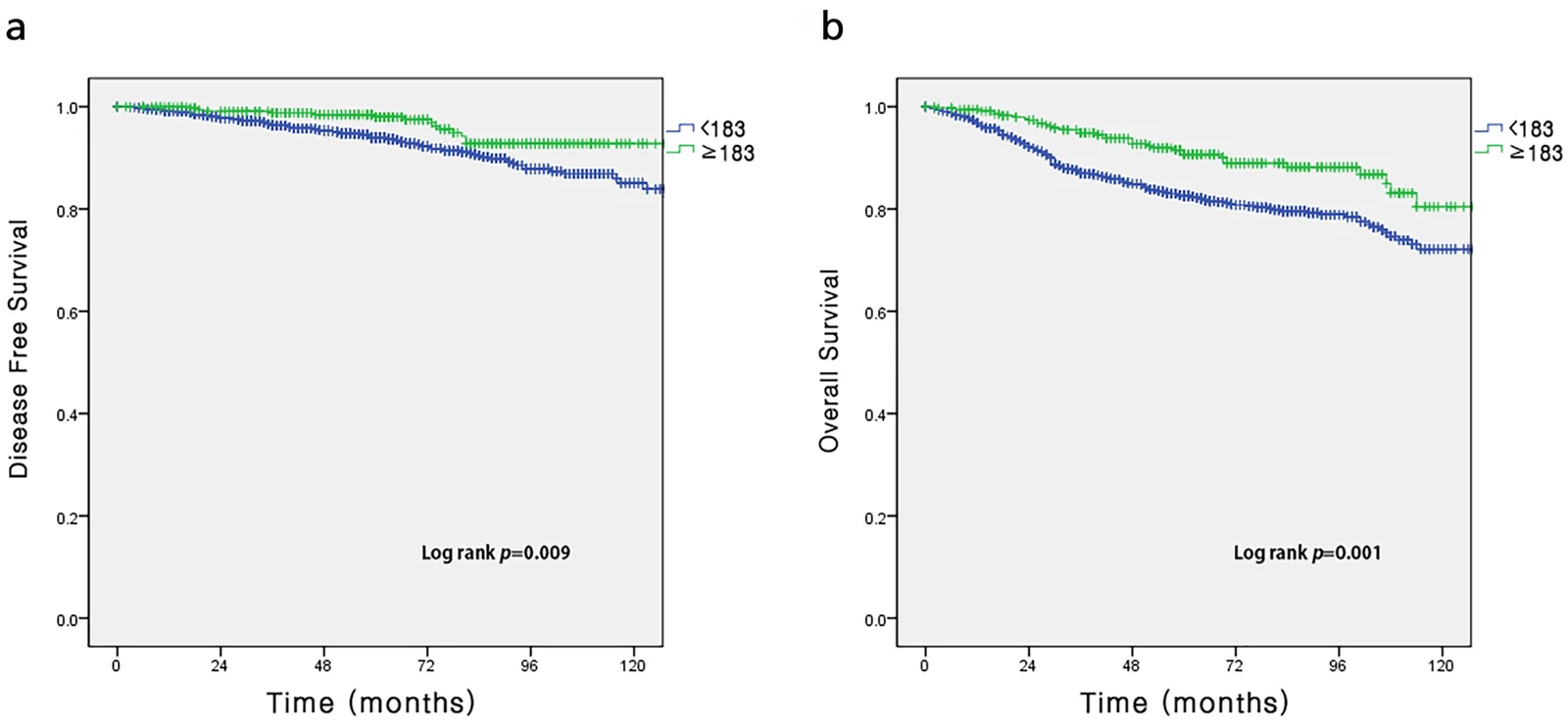
Kaplan–Meier curves of disease-free survival and overall survival for patients with luminal type breast cancer who received neoadjuvant chemotherapy (cutoff value = 183).

The present cohort was followed up for a median of 63 months (range, 0–165). We performed a Kaplan–Meier analysis (Fig 3) to assess the efficacy of the nomogram in predicting patient prognosis. The high-risk group with a nomogram cutoff score of <183 had significantly lower DFS (p = 0.009) and OS (p = 0.001) compared to that in the low-risk group.

To analyze the influence of NACT on certain clinicopathological factors in DFS and OS, a Cox multivariate analysis was conducted (**Table 4**). The results showed that age at diagnosis, nomogram cutoff value of 183 (hazard ratio, 0.67; 95% CI, 0.462–0.970, P < 0.05), and pathologic T stage (specifically ypT2–4) were significantly correlated with DFS. Ki67, clinical T stage, and histologic grade did not correlate with DFS. Therefore, this nomogram provides a tool to distinguish patients with better prognosis compared to those with worse prognosis. Additionally, the sensitivity of our nomogram was 78.2% and the specificity is 27.1

**Table 4:** Cox multivariate analysis for disease-free survival (DFS) and overall survival (OS).

Factors	DFS			OS		
HR	95% CI	p-value	HR	95% CI	p-value
Age at diagnosis	0.981	0.967-0.996	0.012	1.010	0.989-1.032	0.342
Ki-67 index	1.000	0.999-1.000	0.395	1.000	1.000-1.001	0.436
Cutoff 183	0.670	0.462-0.970	0.034	0.558	0.313-0.995	0.048
Pathologic T			<0.001			0.026
pT0	1(ref.)			1(ref.)		
pT1	1.232	0.576-2.636	0.591	1.212	0.351-4.179	0.761
pT2	2.415	1.122-5.198	0.024	2.827	0.818-9.766	0.100
pT3	2.404	1.045-5.533	0.039	2.914	0.762-11.133	0.118
Pathologic N			0.007			0.017
pN0	1(ref.)			1(ref.)		
pN1	1.412	0.960-2.077	0.079	1.319	0.715-2.432	0.376
pN2	1.687	1.071-2.657	0.024	2.183	1.090-4.373	0.028
Clinical T			0.180			0.899
cT1	1(ref.)			1(ref.)		
cT2	1.465	0.815-2.635	0.202	1.149	0.480-2.752	0.755
cT3	1.156	0.610-2.192	0.654	1.053	0.409-2.709	0.914
Clinical N			<0.001			0.049
cN0	1(ref.)			1(ref.)		
cN1	1.810	1.242-2.637	0.002	2.111	1.150-3.875	0.016
cN2	2.887	1.730-4.819	<0.001	2.259	0.978-5.221	0.057
Histologic Grade			0.015			0.055
Grade1	1(ref.)			1(ref.)		
Grade2	2.235	0.547-9.126	0.263	1.582	0.214-11.695	0.653
Grade3	3.716	0.876-15.965	0.075	3.222	0.412-25.195	0.265

DFS: disease-free survival, OS: overall survival, HR: Hazard Ratio, T: tumor, N: node, P: pathologic, C: clinical.

## Discussion

NACT is the standard of treatment for locally advanced breast cancer, including in patients with luminal type breast cancer. The development of a nomogram with widely accessible clinicopathological criteria to predict pCR in patients with luminal type breast cancer undergoing NACT is a significant advancement in the quest for precision oncology. Previous published studies showed that luminal breast cancer shows a low pCR rate (6–21%), which differs from that of the pCR rate for HER2-enriched and triple negative breast cancer [16,17]. Nevertheless, pCR is a well-established indicator for longer DFS and better OS after NACT in breast cancer, but differs from that of other luminal type tumors [18].

Genomic expression assays, such as the 21-gene Oncotype Dx and 70-gene MammaPrint, have been recommended by the National Comprehensive Cancer Network guidelines to be used in the adjuvant setting for stratifying the need for adjuvant systemic therapy in patients with HR-positive/HER2-negative early-stage breast cancer [19]. In 2020, during the COVID-19 pandemic, to identify patients with low-risk tumor biology who may be candidates for surgical delay during the COVID-19 pandemic, McKelley et al. [20] published a poster in the San Antonio Breast Cancer Symposium in 2020 that compared gene expression results of MammaPrint and BluePrint between core needle biopsy and surgical resection samples. They found that both genomic expression assays used on core needle biopsy specimens are reliable and useful tools to triage patients with early breast cancer preoperatively. Moreover, MammaPrint and BluePrint showed a promising role in tailoring preoperative treatment in early-stage breast cancer [21]. Therefore, the idea of developing a nomogram adopted from MammaPrint emerged. The use of genomic assays is time-consuming and adds an additional cost to healthcare systems. Therefore, over the last few years, researchers have been attempting to develop a nomogram that can predict the efficacy of NACT and screen patients who can benefit from chemotherapy [22,23]. Such nomograms can help in selecting patients who will most benefit from NACT. Additionally, it can protect patients from unnecessary chemotherapy toxicity due to non-responsiveness.

Furthermore, Garufi et al. [6] recently published a similar study of 539 patients diagnosed with luminal type breast cancer. The pCR rate in their study was 11.3%. Similarly, they found that clinical stage, high Ki67 score, and PR status were independent predictors of pCR. These data are consistent with previous findings [24]. The present study included 1,574 patients with luminal type breast cancer, which comprises a much larger cohort than the one in the study by Graufi et al.

In this retrospective study, the goal was to validate whether a nomogram that can predict the probability of a low-risk MammaPrint result can predict the response to NACT in women with clinically high-risk breast cancer. We found that the AUC in our study was 0.64, which is an acceptable result that is similar to that reported by Graufi et al. (AUC, 0.7). Additionally, Hwang et al. [15] developed a nomogram that included four variables, including age at diagnosis, nuclear grade, PR Allred score, and Ki67 index in HR-positive/HER2-negative patients in the adjuvant setting. Their nomogram showed satisfactory performance in predicting patients with low genomic risk. The current study adopted this nomogram to predict the MammaPrint risk, and the new nomogram was independently related to the response to NACT.

In the present study, we further validated the nomogram in relation to long-term survival of patients. We performed Kaplan–Meier analysis of high-risk and low-risk groups divided by a nomogram score of 183, which is similar to the cutoff used by Hwang et al. [15], who reported a relevant difference in DFS (p = 0.008) but not in OS (p = 0.056) in the adjuvant setting. In contrast to the study by Hwang et al., the present analysis demonstrated a relevant difference in both DFS (p = 0.009) and OS (p = 0.001). Furthermore, the present study had a longer median follow-up of 63 months compared to 39.4 months in the study by Hwang et al. During the multivariate analysis, Higher cT stages and cN+ in addition to a strong positive ER and weak positive PR and High Ki67 index were strong predictors for clinical response. These values all together in addition to the nomogram we used will help in stratifing those who will benefit from NACT.

The current study has several limitations that should be addressed. First, it is a retrospective study, which may include selection bias. However, we addressed this limitation by clearly identifying our inclusion and exclusion criteria for the study population and including a sample size that was larger compared to previous studies. Second, the present study was conducted in a single center in South Korea, therefore it may not represent the diversity of patients as most of a similar ethnic group. Despite that our cohort included patients from different age groups and clinical characteristics. Furthermore, different centers may have a variation in reporting PR status and Ki67 levels and can raise an issue in reproducing there. Hence, to validate the clinical utility of this nomogram, a recommendations for future multi-center studies with a more diverse cohort across different geographic regions to ensure the effectiveness of this tool in different clinical settings. Additionally the multicenter studies will allow us to compare between the different healthcare settings which will further strengthen the external validity of the study.

### Conclusion

The development of a nomogram to predict pCR in patients with luminal type breast cancer receiving NACT remains a challenge. The nomogram presented herein demonstrated utility in decision-making regarding NACT in patients with luminal breast cancer who are at a clinically high risk. Future research should be directed to further study and validate this nomogram in diverse and larger cohorts to improve treatment decision-making and patient outcomes in this subset of patients.

## Supporting information

S1 Fig (a) Nomogram to predict low-risk recurrence score of the MammaPrint results and receiver operating characteristic curves of the nomogram. (b) Training group of 306 patients. (c) Validation group of 103 patients. (JPG)

S1 File MMP neo submission. (XLSX)

## Abbreviations

AUC: area under the curve
CI: confidence interval
cN0–2: clinical node stage 0–2
cT1–3: clinical tumor stage 1–3
DFS: disease-free survival
ER: estrogen receptor
HER2: human epidermal growth 2
HR: hormone receptor
N: node
NACT: neoadjuvant chemotherapy
OR: odds ratio
OS: overall survival
pCR: pathologic complete response
PR: progesterone receptor
T: tumor
